# Integration of Choline Chloride-Based Natural Deep Eutectic Solvents and Macroporous Resin for Green Production of Enriched Oil Palm Flavonoids as Natural Wound Healing Agents

**DOI:** 10.3390/antiox10111802

**Published:** 2021-11-12

**Authors:** Mohamad Shazeli Che Zain, Jen Xen Yeoh, Soo Yee Lee, Adlin Afzan, Khozirah Shaari

**Affiliations:** 1 Natural Medicines and Products Research Laboratory (NaturMeds), Institute of Bioscience, Universiti Putra Malaysia, Serdang 43400, Selangor, Malaysia; shazelizain@gmail.com (M.S.C.Z.); daphne.leesooyee@gmail.com (S.Y.L.); 2 Department of Chemistry, Faculty of Science, Universiti Putra Malaysia, Serdang 43400, Selangor, Malaysia; yeohjx@gmail.com; 3 Herbal Medicine Research Center, Institute for Medical Research, National Institutes of Health, Ministry of Health Malaysia, Shah Alam 40170, Selangor, Malaysia; adlinafzan@moh.gov.my

**Keywords:** *Elaeis guineensis* leaves, bioflavonoids, NaDES, optimization, wound repair

## Abstract

Huge quantities of oil palm (*Elaeis guineensis* Jacq.) leaves (OPL) are generated as agricultural biomass from oil palm plantations. OPL are known to contain significant amounts of flavonoids. For maximal exploitation of these valuable antioxidant compounds, an innovative and sustainable extraction method employing natural deep eutectic solvents (NaDES) combined with ultrasonic assisted extraction was developed. Various NaDES composed of choline chloride as the hydrogen bond donor (HBD) and 1,2 propanediol (PD), 1,4 butanediol (BD), glycerol (GLY), glucose (GLU), maltose (MAL), and lactic acid (LA) as the hydrogen bond acceptor (HBA) were synthesized. The influence of these compositions, the methods of their synthesis, molar ratios, and water contents on their capacity to extract flavonoids from OPL was evaluated. Based on the results, it was found that methods which incorporate a heating step produced NaDES with the best capacity to extract OPL flavonoids. These thermal methods combined with molar ratios of 1:3 or 1:4 and water contents of 17 to 50% were found to be the optimal conditions for preparing NaDES, specifically when applied to the PD, BD, and GLY NaDES. Subsequently, UHPLC-UV/PDA-MS/MS analysis revealed NaDES extracts recovered by macroporous adsorption resin XAD7HP were able to optimally extract at least twelve luteolin and apigenin derivatives in OPL NaDES extracts prepared from glycerol and 1,4-butanediol demonstrated better and comparable efficiency as aqueous methanol in extracting flavonoids from OPL. The in vitro studies of antioxidant and wound healing properties supported these findings by exhibiting good free radical scavenging, cell proliferation, and migration activities. Additionally, the NaDES extracts also showed non-cytotoxicity effects at 1000 µg/mL and below on 3T3 fibroblast cells. Results of the study showed that NaDES could be a promising eco-friendly green solvent to extract bioactive OPL flavonoids that have great potential for applications as wound healing agents.

## 1. Introduction

Natural deep eutectic solvent (NaDES) is a generation of a new and revolutionary green solvent developed from the principle of ionic liquids (ILs) and deep eutectic solvents (DES) [1]. In formulatng a NaDES mixture, two or more primary plant-based metabolites such as organic acids, alcohols, amino acids, or sugars are combined, acting as a pair of hydrogen bond donor (HBD) and hydrogen bond acceptor (HBA), forming a stable liquid under optimal conditions. In comparison with conventional organic solvents (COS), NaDES offer important advantages such as non-volatility, non- or low toxicity, thermal stability, high biodegradability and biocompatibility, low cost, and easy to prepare, as well as being sustainable [2]. NaDES have found applications in many different areas, particularly in extracting important metabolites from different plant matrices [2,3,4,5,6]. To a certain extent, from the perspective of phytomedicine development, this capacity can help enhance the bioactivity of an extract. However, due to the complexity of the biological system, a universal NaDES that is able to extract any type of plant metabolites from any type of plant matrix is still non-existent. To affect an efficient extraction from a particular plant matrix, the optimal combinations of HBA, HBD, and water components of NaDES need to be formulated. Since NaDES have higher viscosities than COS, it is often combined with ultrasonication technology to assist efficient liberation of metabolites from the plant matrix [7,8,9]. The recovery of compounds after extracted by NaDES has not been extensively studied. Nevertheless, several methods such as recrystallization, anti-solvents, macroporous adsorption resin, supercritical carbon dioxide, solid-phase extraction, and preparative high-performance liquid chromatography (HPLC) techniques have been used to evaluate the recovery of extracted compounds from NaDES [2,10,11,12,13,14,15].

Oil palm (*Elaeis guinessis* Jacq.) leaves (OPL) have been established to contain a significant amount of flavonoids and their potential applications have been demonstrated by studies on their biological properties, especially for antioxidant, anti-inflammatory, and wound healing activities [16,17,18,19,20,21,22,23]. In previous studies, we introduced an integrated process of extraction and enrichment of flavonoids from OPL which is simple, rapid, and efficient [16,17]. Although the combination of ultrasound-assisted extraction and use of macroporous resin in the process significantly improved the quality of the extract produced without using excess amounts of COS, the sustainability of the integrated process can be further enhanced by integrating NaDES as the extraction media in the workflow, replacing COS. Moreover, previous studies have reported that NaDES could extract flavonoids from different plant materials as efficiently as COS [4,24,25].

**Figure 1:**
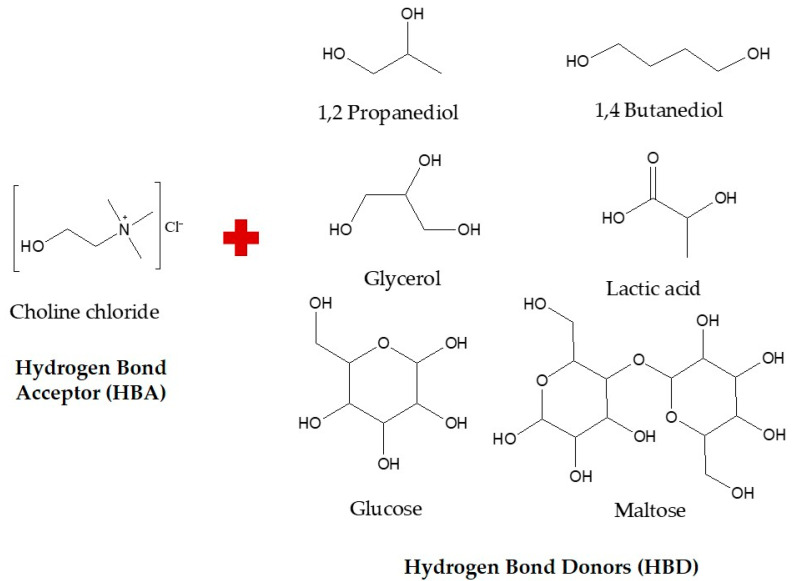
Structures of hydrogen bond acceptor (HBA) and hydrogen bond donor (HBD) used in the present study.

**Figure 2:**
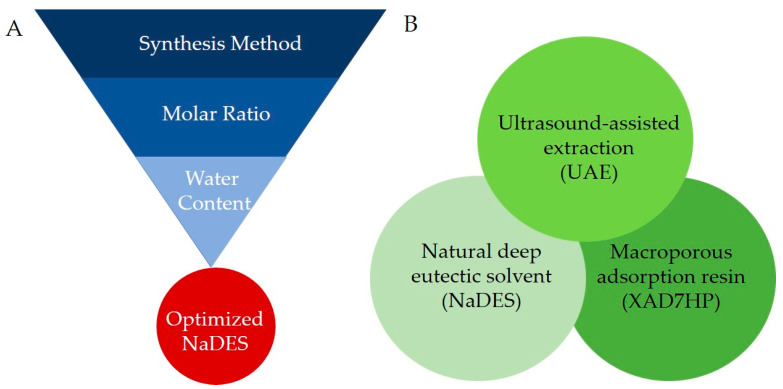
(**A**) General scheme for optimization of NaDES. (**B**) Integration of three key components as a green strategy for flavonoid extraction from oil palm leaves (OPL).

In the present study, we prepared and optimized various compositions of NaDES for the extraction of flavonoids from OPL, and evaluated the feasibility of incorporating it in the aforementioned integrated process as an alternative green solvent in place of COS. Choline chloride was selected as the HBA while six naturally occurring compounds comprising 1,2 propanediol, 1,4 butanediol, glycerol, glucose, maltose, and lactic acid were used as the HBD (Figure 1) [26]. The method of synthesis, molar ratio, and water contents of the NaDES were optimized for maximum performance in extracting OPL flavonoids which was assessed based on total flavonoid content (TFC) of the extract produced (Figure 2A). Subsequently, extracts obtained using the optimal choline chloride-based NaDES formulation were recovered using macroporous resin XAD7HP and further chemically profiled using ultra-high-performance liquid chromatography ultraviolet visible/photodiode array tandem mass spectrometry (UHPLC-UV/PDA-MS/MS). To ascertain the quality and potential biological applications of the end product obtained through the integrated process, the antioxidant and wound healing activities of the NaDES extracts were also evaluated.

## 2. Materials and Methods

### 2.1. Chemicals, Reagents, and Equipment Used in This Study

Choline chloride, lactic acid, malic acid, 1,4-butanediol, 1,2-propanediol, glycerol, glucose, maltose, Amberlite XAD7HP, sodium acetate, phosphoric acid, quercetin, sulfanilamide, and *N*-(1-naphthyl) ethylenediamine dihydrochloride were purchased from Sigma (Aldrich, Germany). Aluminum chloride was provided by HmbG Chemicals (Hamburg, Germany). Ethanol and methanol were obtained from R&M Chemicals (Essex, UK) while sodium nitroprusside was supplied by Bendosen Laboratory Chemical (Bendosen, Norway). Hydrochloric acid, dimethyl sulfoxide (DMSO), sodium hydroxide, and LCMS grade acetonitrile were purchased from Merck (Darmstadt, Germany). Purified water from MilliQ system (Millipore, Bedford, MA, USA) was used throughout the study. Vitexin, isovitexin, orientin, and isoorientin with purity higher than 98.0% were supplied by ChemFaces (Wuhan, China).

Dulbecco’s modified eagle medium (DMEM), phosphate buffer saline (PBS) solution, and thiazoyl blue tetrazolium bromide (MTT) were purchased from Solarbio (Beijing, China) while penicillin-streptomycin and trypsin were from Nacalai Tesque (Kyoto, Japan). Fetal bovine serum was supplied by Tico Europe (Amstelveen, Netherlands), while 3T3 mouse fibroblasts were obtained from Cell Line Service (Appelheim, Germany).

A Philips mechanical grinder (HR2056, Eindhoven, Netherlands) was used for preparation of powdered leaf material. A Heidolph vacuum rotary evaporator (Heidolph Instruments GmbH and Co.KG, Schwabach, Germany) was used for vacuum evaporation, and a Labconco^®^ FreeZone Freeze Drier System (Kansas City, MO, USA) was used for sample freeze-drying. Other equipment used were magnetic stirrer hotplate (C-MAG HS7, IKA^®^, Wilmington, NC, USA), ultrasonic water bath (Branson 2510MT Ultrasonic Cleaner, Darmstadt, Germany), and shaking incubator (WiseCube WIG-10RL Precise Shaking Incubator, Wisd Laboratory Instruments, Wertheim, Germany).

### 2.2. Sample Preparation

OPL sample preparation followed our previously established method with minor modifications [17]. Briefly, the leaflets were cut into one inch length and freeze-dried at −50 °C until constant weight. The dry material was further pulverized and passed through 300 µm pore size sieve to obtain uniform particle powder. The prepared sample was then stored at −80 °C prior to use.

### 2.3. Preparation and Optimization of NaDES Formulation

Six formulation of choline chloride-based NaDES were prepared with the HBDs 1,4 butanediol, 1,2 propanediol, glycerol, glucose, maltose, and lactic acid, labeled respectively as BD, PD, GLY, GLU, MAL, and LA. Three main parameters in the synthesis of the choline chloride-based NaDES, namely the method of synthesis, molar ratio, and water content, were examined. The effect of these parameters on the extraction of total flavonoids from OPL was evaluated using a single factor experimental approach in which one factor was tested at a time, while other factors remained unchanged. The various NaDES components were weighed according to the combinations of required molar ratios and water contents, and placed in 5 mL screw-capped glass vials containing a stirring bar magnet of the appropriate size [2]. The various NaDES produced were evaluated for homogeneity (based on physical appearance) and OPL flavonoid extraction efficiency (based on TFC).

#### 2.3.1. Method of Synthesis

Six different synthesis methods were selected for evaluation. Three methods involved application of heat, comprising of stirring with direct heating (SDH), stirring water-bath heating (SWB), and stirring with direct heating followed by sonication (SDS). No heating was used for the remaining three methods, comprising of stirring (SWH), stirring followed by vacuum evaporation (SVD), and stirring followed by freeze-drying (SFD). The molar ratio was fixed at 1:1 (1 molar of HBA and 1 molar of HBD) and the water content at 29%. Three replications were made for each method. For the non-thermal methods (SWH, SVD, and SFD), the experiment was carried out at ambient temperature, with continuous stirring for 30 min on a magnetic stirrer hotplate, set to 800 rpm. While stirring, the water component was added dropwise to facilitate solvation. For the SVD method, the final mixture was subjected to vacuum evaporation with the temperature maintained at 50 °C, while for the SFD method, the final mixture was freeze-dried. For the SDH, SWB, and SDS methods, the mixtures were continuously stirred (800 rpm) for 30 min at 60 °C. Similarly, water was added dropwise while continually stirring the mixture. For the SWB method, the glass vial containing the mixture was placed in a 50 mL glass beaker half-filled with water preheated to 60 °C. For the SDS method, after 30 min of direct heating, the mixture was subjected to sonication (40 Hz) for 15 min at 40 °C. All the prepared NaDES, kept dry in desiccators, were subjected to analysis to determine the best method of synthesis.

#### 2.3.2. Molar Ratios

After determination of the best method of synthesis as mentioned above, the single factor optimization was continued using the selected method for varying molar ratios. The molarity of choline chloride was kept constant while the molarity of the HBD components (1,4 butanediol, 1,2-propanediol, glycerol, lactic acid, malic acid, glucose, and maltose) were each varied to give 5 different molar ratios (1:1, 1:2, 1:3, 1:4, and 1:5). The water content was maintained at 29% to facilitate the dissolution of NaDES. Similarly, all the prepared NaDES were subjected to analysis to determine the best molar ratio.

#### 2.3.3. Water Contents

After determination of the optimal molar ratio, the single factor optimization was continued varying the amounts of the water component (17 to 50%), while maintaining the optimal method synthesis and molar ratio. Calculation of the water content (W) was based on the formula W = (W_water_/100 × W_HBA+HBA_), where W_HBA+HBD_ is the total weight of HBA and HBD (g) while W_water_ is the weight of water added into the formulation (g). The water content is presented as percentage (%).

### 2.4. Ultrasound-Assisted Extraction of OPL with Optimized NaDES

OPL powder (0.02 g) was mixed with 2 mL NaDES in 2 mL microcentrifuge tube. The mixture was vortex-mixed at 3000 rpm for 30 s, and then subjected to sonication (40 Hz) for 30 min at 25 °C. The mixture was then centrifuged at 4000 rpm for 15 min, after which an aliquot amount of the supernatant was sampled and analyzed for TFC. The extraction was performed in triplicate.

### 2.5. Analysis of Compound Recovery from Macroporous Resin

Recovery of the total flavonoids was conducted in a 250 mL scotch bottle containing 140 mL supernatant (BD, PD, GLY, GLU, MAL, LA, and MEOH) with an accurately weighed XAD7HP (4.4 g). The adsorption of the phytochemicals onto pretreated resin was shaken continuously in the set to shaking speed of 250 rpm, at 25 °C, for 24 h. After reaching adsorption equilibrium, the supernatant was carefully discarded and the resin was rinsed with deionized water. Then, 140 mL 95% aqueous ethanol was added into the scotch bottle containing the resin. The shaking was continued at the shaking speed of 250 rpm, at 25 °C, for another 24 h. The supernatant was filtered, collected, and kept under cold condition (4 °C). The adsorption and desorption process were repeated three times to obtain maximum yield. The combined supernatant was vacuum evaporated followed by solid-phase extraction (SPE) to remove residual NaDES which, due to its non-volatility, trace amounts of it were still present after vacuum evaporation. Briefly, for the SPE, C_18_ cartridge was placed in a vacuum manifold and equilibrated with 5 mL ethanol, followed by 5 mL deionized water. Then, a 1 mL sample was loaded onto the cartridge which was subsequently rinsed with 6 mL deionized water. The extract was recovered with the elution of 6 mL of ethanol. After evaporation of the solvent, the extract was freeze-dried, weighed, and subjected to analysis.

### 2.6. Determination of Total Phenolic Content

Total phenolic content (TPC) of the various NaDES extracts was determined using Folin–Ciocalteu reagent as previously described [19]. Briefly, the sample was prepared in methanol as 0.1 mg mL^−1^ solution. Next, 20 µL of the sample was mixed with 100 µL Folin–Ciocalteu reagent in a 96-well plate, and 80 µL of 7.5% sodium carbonate solution was added after 5 min incubation. The 96-well plate was covered and further incubated in the dark for 30 min, after which the absorbance was measured at 750 nm. The analysis was done in triplicates, and the results expressed in milligrams of gallic acid equivalents per gram of dried extract (mg GAE g^−1^ dried extract).

### 2.7. Determination of Total Flavonoid Content

Analysis of the total flavonoid content (TFC) was performed using aluminum chloride complex forming assay as previously reported [17]. Briefly, 125 µL of the samples was mixed with 375 µL 95% ethanol, 25 µL 10% aluminum chloride solution, 25 µL 1 M sodium acetate solution, and 700 µL distilled water in 2 mL microcentrifuge tube. The mixture was vortex-mixed and incubated at 25 °C for 40 min, after which the absorbance was measured at 415 nm. The analysis was done in triplicates, and the results expressed in milligrams of quercetin equivalents per gram of dried extract (mg QCE g^−1^ dried extract).

### 2.8. Determination of Antioxidant Free Radical Scavenging Activity

The 1,1-diphenyl-2-picrylhydrazyl (DPPH) and nitric oxide (NO) free radical scavenging activity assays were performed as described earlier [17]. For DPPH assay, 0.1 mg extract was weighed and transferred into 1 mL methanol, and serially diluted to concentrations ranging from 100 to 0.7 µg mL^−1^. Each test concentration (50 µL) was then mixed with 100 µL DPPH (5.9 mg 100 mL^−1^) in a 96 well microtiter plate and incubated in the dark for 30 min, after which the absorbance was measured at 515 nm.

For NO assay, 1 mg extract was weighed and transferred into 1 mL dimethyl sulfoxide (DMSO), followed by dilution with DMSO to reach final concentrations ranging from 1000 to 0.98 µg mL^−1^. Each test concentration (60 µL) was mixed with 60 µL sodium nitroprusside (0.05236 g dissolved in 20 mL 10 mM phosphate buffer saline) in a 96 well microtiter plate, and incubated at 25 °C for 150 min. After incubation, 60 µL Griess reagent (0.1 g sulphanilamide and 0.01 g *N*-(1-naphthyl) ethylenediamine dihydrochloride in 10 mL 2.5% phosphoric acid) was added into each well and the absorbance measured at 550 nm. Measurements of absorbance of both assays were carried out using Tecan Infinite F200 Pro plate reader (Tecan Group Ltd., Männedorf, Switzerland). The scavenging activity (SA) was calculated according to the formula SA% = ((A_o_ − A_s_)/A_o_) × 100%, where A_o_ and A_s_ are the absorbance values of the blank and test samples, respectively. Quercetin was used as positive control. The analysis was done in triplicates and the results are expressed as percentage of inhibition (%).

### 2.9. Determination of Cell Proliferation and Migration Activity

3T3 mouse fibroblasts were maintained in complete Dulbecco’s Modified Eagle Medium (DMEM) high glucose media with 10% fetal bovine serum (FBS) and 5% of penicillin-streptomycin in a humidified 5% CO_2_ incubator at 37 °C. The media was changed every 48 h. Cells at 70–80% confluence were regarded as ready for seeding and treatment. Cell viability assay was first evaluated by MTT assay, according to a previously described method [16]. Different concentrations of the test samples were prepared, comprising 1.56, 3.13, 6.25, 12.5, 25, 50, and 100 µg mL^−1^. 3T3 cells were cultured in 96-well culture plates at density 1 × 10^5^ cells/well with DMEM complete media for 24 h. The medium was then replaced with 100 µL test sample of the different concentrations, and incubated for 48 h. A 5 mg mL^−1^ MTT solution was prepared in phosphate-buffered saline (PBS), and 20 µL aliquots of the solution were added into each sample well. The plates were incubated for a further 4 h at 37 °C. Finally, the medium was replaced with 100 μL DMSO and the absorbance of the test mixture in each well was measured at 570 nm. Analysis was performed in triplicates. Allantoin was used as a positive control drug, while untreated cells served as negative control.

The cell proliferation and migration were examined using the scratch assay method [16]. 3T3 cells were seeded in 24-well plates at a density of 3 × 10^5^ cells/well and allowed to grow for 24 h at 37 °C and 5% CO_2_. A small linear scratch of uniform size and distance was made on the confluent monolayer by carefully scraping with a sterile P200 pipette tip. Cells were then rinsed with PBS (thrice) for complete removal of cellular debris before adding the media with different treatment solutions at different concentrations (1.56, 3.13, 6.25, 12.5, 25, and 50 µg mL^−1^). The wound closures were monitored and photographed by phase contrast miscroscopy using ×5 magnification at 0 h. After 24 and 48 h of incubation, the recorded images were analyzed using Image-J software (Bethesda, MD, USA) and percentage of the closed area was measured and compared to the value obtained before treatment. An increase of the percentage of closed area indicated the migration of cells. Allantoin was used as a positive control drug and cells without treatment were used as negative control. All scratch assays were performed in six replicates.

### 2.10. UHPLC-UV/PDA-MS/MS Analysis

The OPL extract was separated using C_18_ reversed-phase Acquity UPLC^®^ BEH 1.7 µm particle size and 2.1 mm i.d. × 100 mm length column from Waters (Dublin, Ireland) on Thermo Scientific Ultimate 3000 (Dionex, Sunnyvale, CA, USA) with a PDA-3000 photodiode array detector and a thermostat column compartment which was maintained at 40 °C during UHPLC analysis. Gradient elution was performed with water:0.1% formic acid (solvent A) and acetonitrile:0.1% formic acid (solvent B) as mobile phase. The programmed gradient proceeded using following sequence of solvent B percentages: 5% for 0–15.0 min, 5–20% for 15.0–27.0 min, 95% for 27.0–30.0 min, 95–5% for 30.0–31.0 min, and 5% for 31.0–38.0 min. The flow rate was constant at 0.30 mL/min. The UV detector was set at 254, 280, and 366 nm. The chromatographic peaks were identified by comparing their respective retention times and UV/PDA spectra with those of reference standards, which were eluted in parallel under the same conditions. All samples were prepared 5 mg mL^−1^ and filtered through a 0.22 µm syringe filter (Sartorius AG, Goettingen, Germany) for UHPLC injection. The regression curves for orientin and vitexin were *y* = 2706.2*x* − 19677 and *y* = 534.05*x* − 6500.5, respectively, where y is the peak area of the compound and x is the concentration of the respective standards. The results of total luteolin (TLC) are expressed in milligrams of orientin equivalents per gram of extract (mg OE g^−1^ dried extract) while the results of total apigenin (TAC) are expressed in milligrams of vitexin equivalents per gram of extract (mg VE g^−1^ dried extract). The total flavonoid C-glycoside (TFCGC) is expressed as a result of addition of total apigenin and luteolin (mg g^−1^ dried extract).

The MS analysis was done on Q-Exactive Focus Orbitrap LC-MS-MS system (Thermo Fisher Scientific, San Jose, CA, USA). The eluent was monitored by ESI-MS under negative mode scanned from *m/z* 67.9 to 1000. ESI was conducted using a spray voltage of 4.2 kV. High purity nitrogen gas was used as dry gas at a sheath gas flow rate of 40 (arbitrary units) and aux gas flow rate of 8 (arbitrary units). Capillary temperature was set at 320 °C while aux gas heater temperature was set at 0 °C. For tandem MS (MS/MS), data acquisitions were carried out in the automatic mode at 30 eV collision energy. Data acquisition and processing were performed by Thermo Xcalibur Application version 2.2 by Thermo Fisher Scientific (San Jose, CA, USA).

An adaptation of conventional nomenclature [27,28] is used in this work to explain fragment ions of glycoconjugates. Fragment ions denoted as *^k,l^_j_*X*_n_* represent ions still comprising the flavone aglycon with *k* and *l* denoting the cleavage positions within the carbohydrate rings. The total carbon in the monosaccharide interglycosylated to aglycon (hexose, 6; pentose, 5) is represented by *j* while *n* refers to the attachment position of the saccharide to the aglycon (C-6 or C-8).

### 2.11. Statistical Analysis

For quantitative analysis of polyphenolic contents (TPC and TFC), luteolin and apigenin derivatives, antioxidant free radical scavenging (DPPH and NO), and cell proliferation and migration activities, Minitab (Minitab Inc, State College, PA, USA) and GraphPad statistical software (San Diego, CA, USA) were applied. The values are shown as a mean ± standard deviation. To determine the significant differences of the values obtained, one-way analysis of variance (ANOVA) followed by Tukey’s test was performed, where *p* < 0.05 was set as the significant level. In addition, the correlation between luteolin and apigenin derivatives with the antioxidant and wound healing activities were examined with partial least square analysis (PLS) model, performed using Pareto scaling using version 13.0 SIMCA software (Umetrics AB, Umea, Sweden).

## 3. Results and Discussion

### 3.1. Optimization of Choline Chloride-Based NaDES

#### 3.1.1. Method of Synthesis

**Figure 3:**
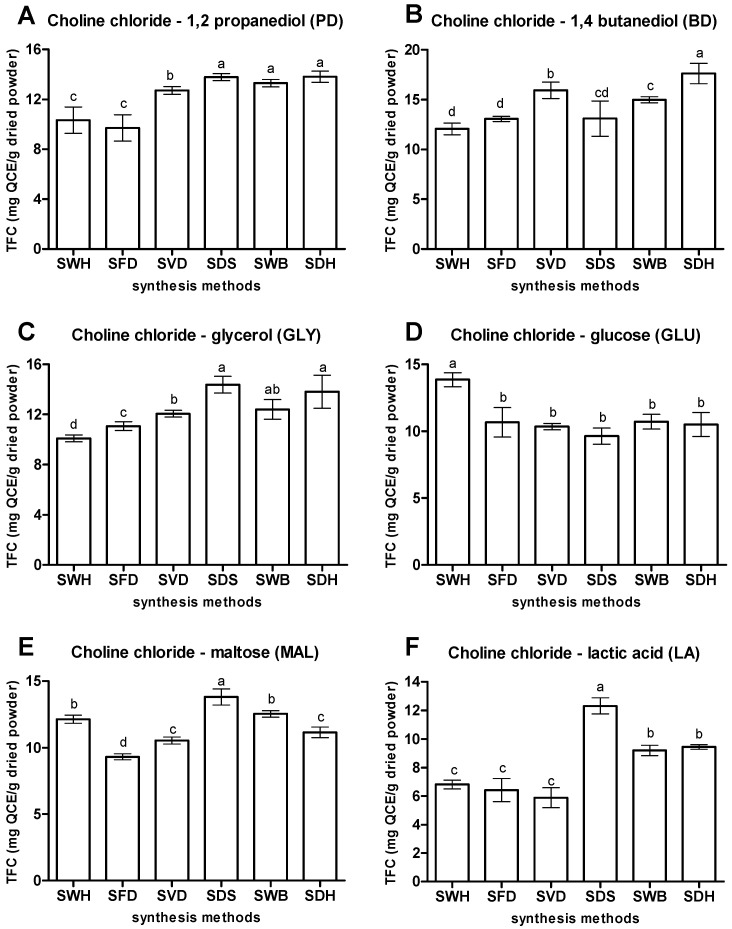
Effect of different synthesis methods of NaDES. (**A**) Choline chloride—1,2 propanediol, (**B**) Choline chloride—1,4 butanediol, (**C**) Choline chloride—glycerol, (**D**) Choline chloride—glucose, (**E**) Choline chloride—maltose and (**F**) Choline chloride—lactic acid. Values are presented as mean ± standard deviation of three measurements. SDH—stirring with direct heating; SWB—stirring water-bath heating; SDS—stirring with direct heating followed by sonication; SWH—stirring without heating; SVD—stirring and vacuum evaporation; SFD—stirring and freeze-drying. Values marked with different letters indicate comparison among synthesis methods in NaDES which are statistically significant differences at *p* < 0.05.

Figure 3 shows the TFC of extracts produced by the various choline chloride-based NaDES prepared using different synthesis methods. PD prepared using the thermal methods (SDS, SWB, and SDH) showed better capacity of extracting OPL flavonoids. The highest TFC was obtained for PD prepared via SDH (13.82 mg QCE/g), although the value was not statistically different from those obtained from PD prepared via SDS and SWB. Similarly for BD, the highest TFC was obtained for BD prepared via SDH (17.62 mg QCE/g), which was significantly higher than those obtained from BD prepared via SDS and SWB. It was also observed that NaDES prepared via SDH rapidly produced a clear liquid compared to the other methods of synthesis. It has been reported that the formation of homogenous NaDES resulted from the increment of entropy of the system which enhanced the eutectic formation in the presence of heat during synthesis process [29]. Other researchers have also used heat in preparing their respective NaDES formulation during the synthesis process [2,10,25,30,31].

Enhancement of the NaDES capacity for extraction of phytochemicals can be improved by introducing ultrasonic energy during the NaDES preparation. This was observed for the NaDES GLY, MAL, and LA prepared by SDS which produced extracts with the highest TFCs of 14.38, 13.81, and 12.32 mg, respectively. The involvement of ultrasonication in the synthesis of NaDES cavitated the system by releasing energy which further initiates the formation of intermolecular forces between the components [32]. Recently, ultrasonication has become a method of choice in preparing NaDES, since it is not only effective, simple to use, and low cost, but also speeds up the whole extraction process [12,15,32].

On the other hand, for the glucose containing NADES, GLU, the highest TFC (13.85 mg QCE/g), was obtained for GLU prepared without application of heat (SWH). GLU prepared using other methods produced TFC lower than 10.0 mg QCE/g) with no significant differences between them. Thus, it was hypothesized that combination of heating and ultrasonication in the preparation of the various GLU may have provided excessive energy leading to disruption of hydrogen bonds, resulting in NaDES with lower capacities for extracting the flavonoids. However, although the choline chloride–glucose mixture can be prepared non-thermally, it was also observed that it took a much longer time for the solid-state components to form a homogenous liquid, even with continuous stirring.

In general, thermally prepared choline chloride-based NaDES (SDH, SWB, and SDS) exhibited better capacity to extract OPL flavonoids compared to non-thermally prepared NaDES (SWH, SFD, and SVD), except for the choline chloride-glucose NaDES combination. Ultrasonication also effected additional improvements to the final results. Thus, based on their optimal extraction capacities, PD (SDH), BD (SDH), GLY (SDS), MAL (SDS), LA (SDS), and GLU (SWH) were selected for use in subsequent experiments, discussed in the following sections.

#### 3.1.2. Molar Ratios

**Figure 4:**
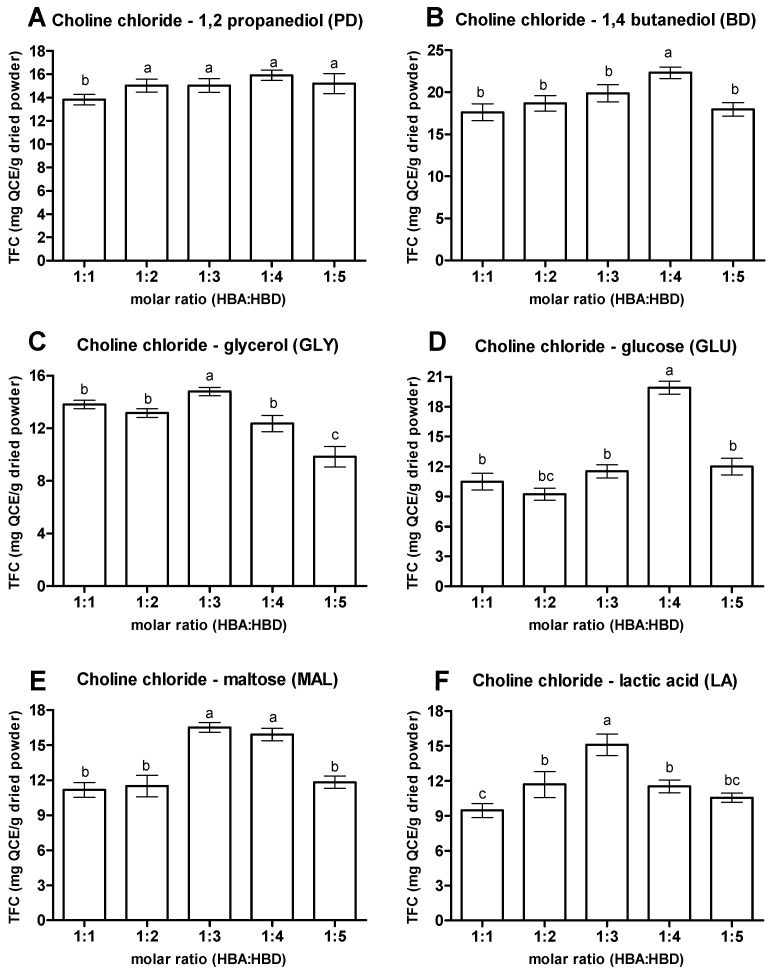
Effect of different molar ratios of NaDES. (**A**) Choline chloride—1,2 propanediol, (**B**) Choline chloride—1,4 butanediol, (**C**) Choline chloride—glycerol, (**D**) Choline chloride— glucose, (**E**) Choline chloride—maltose and (**F**) Choline chloride—lactic acid. Values are presented as mean ± standard deviation of three measurements. Values marked with different letters indicate comparison among molar ratios in NaDES which are a statistically significant differences at *p* < 0.05. HBA, hydrogen bond acceptor; HBD, hydrogen bond donor.

Molar ratio of HBA to HBD plays a significant role in developing an efficient NaDES. Figure 4 shows the TFC of extract obtained from the various NaDES prepared in varying molar ratios, where different efficiency in extracting flavonoids from OPL was observed. However, despite the different HBD in the NaDES composition, there was a trend observed for the TFC extracted by the NaDES mixed at different molar ratios, where the TFC extracted increased with the increase of molar ratio of HBD, before a decline happened in the maximum yield of TFC. For the NaDES with 1,2 propanediol as the HBD (PD), though the highest extraction efficiency for TFC was obtained when the NaDES was prepared with molar ratio of 1:4 (TFC 15.91 mg QCE/g), the TFCs extracted were actually not significantly different when the molar ratios of NaDES increased from 1:2 to 1:5. For the NaDES containing glycerol (GLY) and lactic acid (LA), the highest TFC was obtained at the molar ratio of NaDES at 1:3, with TFC values of 14.80 and 15.09 mg QCE/g, respectively. On the other hand, molar ratio of 1:4 was the optimal one of NaDES components for the 1,4 butanediol and glucose containing NaDES (BD and GLU), while NaDES with maltose as HBD exhibited the highest TFC extraction efficiency at the molar ratios of 1:3 and 1:4, with TFC values of 16.50 and 15.90 mg QCE/g, respectively, which had no significant differences among them. Thus far, the results suggested that the presence of additional hydroxyl groups in the mixtures contributed by higher amounts of HBD (1,2 propanediol, 1,4 butanediol, and glycerol) increased the chances of formation of more hydrogen bonds with compounds of interests which ultimately increased the extractability competency [2].

Nevertheless, excessive addition of HBD into the NaDES composition may have a negative effect on the extractability of NaDES for flavonoids from OPL. The low TFC value of all the NaDES prepared at 1:5 molar ratio indicated the disproportionate amount of HBD components (1,2 propanediol, 1,4 butanediol, glycerol, glucose, maltose, and lactic acid) with HBA (choline chloride), which resulted in an inefficient NaDES for flavonoid extraction. This could be due to the steric hindrance phenomenon between the excessive components in the mixtures that interrupted the hydrogen bonding interactions [31,33]. Overall, the present study suggests that 1:3 and 1:4 were the optimal molar ratios of choline chloride (HBA) and the studied HBDs for extracting flavonoid from OPL.

#### 3.1.3. Water Contents

**Figure 5:**
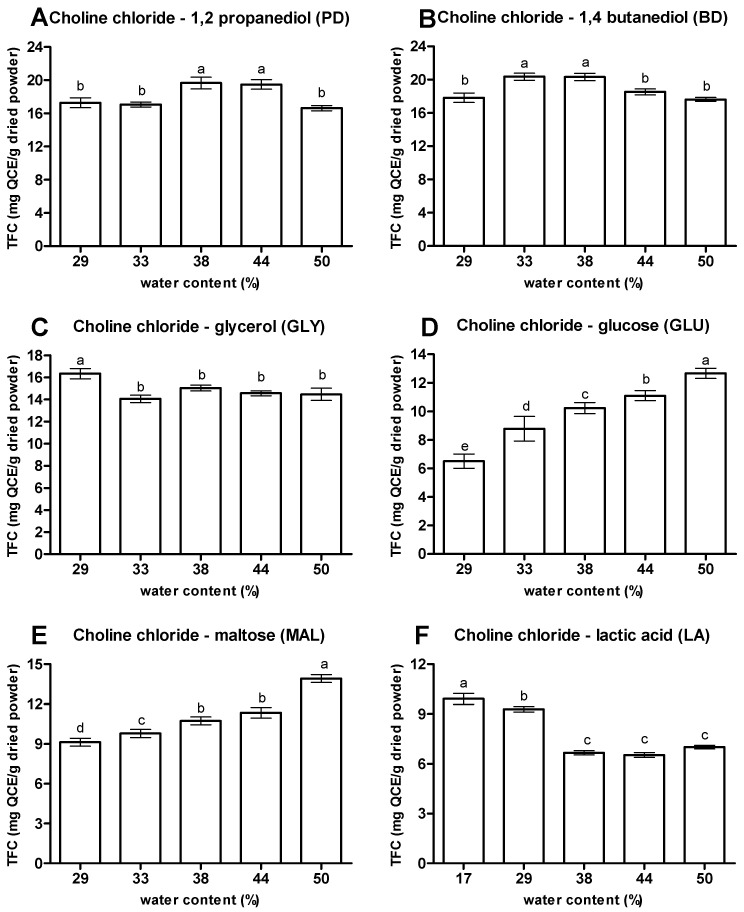
Effect of different water contents of NaDES. (**A**) Choline chloride—1,2 propanediol, (**B**) Choline chloride—1,4 butanediol, (**C**) Choline chloride—glycerol, (**D**) Choline chloride—glucose, (**E**) Choline chloride—maltose and (**F**) Choline chloride—lactic acid. Values are presented as mean ± standard deviation of three measurements. Value marked with different letters indicate comparison among water contents in NaDES which are statistically significant differences at *p* < 0.05.

The evaluation of the effect of addition of water in NaDES on its ability to extract flavonoids from OPL is shown in Figure 5. In agreement with previous studies [10,34], the addition of water (17 to 50%) in the NaDES system significantly modified the viscosity of the prepared NaDES, thus influencing the mass transfer system between NaDES and the compound of interest. The NaDES with 1,2 propanediol (PD) was able to extract TFC ranging from 16.64 to 19.67 mg QCE/g, where 38% and 50% extracted the highest and lowest TFC, respectively. A similar trend was shown by NaDES with 1,4 butanediol (BD) where 33% and 50% were able to extract the highest and lowest TFC with values of 20.36 and 17.62 mg QCE/g, respectively. For NaDES with glycerol (GLY) and lactic acid (LA), GLY (29%) and LA (17%) showed the highest TFC with values of 16.34 and 9.92 mg QCE/g, respectively. The study indicated that though the high-water content enhanced handling and improved the mass transfer, the excessive water content added into the system affected the supramolecular structures of developed NaDES by significantly increasing the deformation of hydrogen bonds between NaDES components [1,2,12,15,34,35,36].

Unlike the trend shown by other NaDES, NaDES with glucose (GLU) and maltose (MAL) experienced a linear increase of TFC values with the rise of the water component. Both GLU and MAL with 50% water content were able to extract the highest TFC with values of 12.67 and 13.92 mg QCE/g, respectively. With gradual increase of water into the mixture, the efficiency of sugar based NaDES in extracting flavonoids from OPL was improved. It was observed at a low amount of water that the viscosity of the mixtures was poor, which contributed to negative impacts on the efficiency of the mass transfer mechanism of compounds of interest from the interior of plant material to the NaDES [5,10,37].

To summarize, the studies on the effects of synthesis methods, molar ratios, and water contents on NaDES for optimal flavonoid extraction from OPL revealed PD (SDH, 1:4, 38%), BD (SDH, 1:4, 33%), GLY (SDS, 1:3, 29%), GLU (SWH, 1:4, 50%), MAL (SDS, 1:3, 50%), and LA (SDS, 1:3, 17%) were the optimal conditions. Hence, these NaDES were further used as solvent for ultrasonic-assisted extraction (UAE) of OPL flavonoids and integrated with an enrichment step using macroporous (XAD7HP) resin. The recovered NaDES extracts were subsequently subjected to chemical and biological evaluations, discussed in the following sections.

### 3.2. Identification and Quantification of Flavonoids in OPL–NaDES Extracts

The extracts produced by the various optimized NaDES were further chemically profiled using UHPLC-UV/PDA-MS/MS analysis. As shown in Figure S1 and Table 1, a total of twelve flavonoids, consisting of six luteolin derivatives (peaks 1, 3, 5, 6, 7, and 9) and six apigenin derivatives (peaks 2, 4, 8, 10, 11, and 12) were putatively identified and relatively quantified in the NaDES extracts. The identification of these compounds was assisted by published results [16,17,20,21] and comparison with pure standards.

Peaks 1 and 3, both with *m/z* 609.1411, showed fragments at *m/z* 519.1104, 489.0998, 429.0786, 399.0696, and 369.0585 for losses of 90, 120, 180, 210, and 240 amu, respectively. Fragmentation of C-hexosyl conjugate at the 6-C position of luteolin (aglycon; Agly) resulted in [^0,3^_6_X_6_]^−^ ion at *m/z* 519.1104 for [(M-H)-90]^−^, and further cleavage of the hexose produced [^0,2^_6_X_6_]^−^ ion at *m/z* 489.099 for [(M-H)-120]^-^ (Figure S2). The presence of [Agly+83]^−^ and [Agly+113]^−^ ions is most probably due to the fragmentation of both 6-*C* and 8-*C* hexosyl with breakages at 0 and 3” positions for both hexoses to give *m/z* 399.0696, and further loss of CH_2_O moiety from any of the hexosyl ring to give *m/z* 369.0616. An abundance of the *m/z* 369.0585 and 399.0696 ions in the MS/MS spectra for peaks 1 and 3 is significant for the existence of distinctive [Agly+83]^−^ and [Agly+113]^−^ ions in C-glycosylated flavones. This is in agreement with previously published results [20,38,39], indicative of the presence of two C-glycosylated sugars to the flavone moiety, which, in this case, is luteolin (C_18_H_10_O_6_, MW 286). Therefore, peaks 1 and 3 were identified as luteolin-6,8-di-C-hexose isomers.

Peaks 5 and 6 exhibited molecular ions at *m/z* 447.0896 [M-H]^−^ indicating that they have the same molecular formula of C_21_H_20_O_11._ Both peaks showed similar fragmentation pattern with compounds 1 and 3, where fragment ions at *m/z* 357.0588 and 327.0483, for the losses of 90 and 120 amu, respectively, were consistently generated with high relative abundance. In addition, the appearance of *m/z* 285.0381 [(M-H)-162]^−^, indicating the loss of a hexose unit from the aglycone moiety. Therefore, peaks 5 and 6 were assigned as isoorientin (luteolin-6-C-glucoside) and orientin (luteolin-8-C-glucoside), respectively, and confirmed by comparing with analytical standards.

Peaks 7 and 9 also have similar molecular ions at *m/z* 593.1464. The fragmentation generated [(M-H)-120]^-^ ions *m/z* 473.1049 for loss of a hexose moiety. Further fragmented produced ions at *m/z* 429.0792, 369.0590 and 327.0485 for [(M-H)-44]^−^, [(M-H)-44-30]^−^, [(M-H)-44-60]^−^ and [(M-H)-44-90]^−^, respectively, ions that are characteristic of *C*-deoxyhexose fragmentation [40]. Based on these data, the peaks were identified as those of luteolin-*C*-hexose-C-deoxyhexose isomers, which was further supported by the presence of fragment ions at *m*/*z* 357.0589 ascribable to [Agly+83]^−^ and neutral loss of 120 amu (hexose). Based on relative abundance and previously reported results [20], the specific position of the hexose and deoxyhexose moieties were deduced, identifying peaks 7 and 9 as luteolin-6-C-hexose-8-C-deoxyhexose isomers.

The consistent presence of ions at *m/z* 383 [Agly+113]^−^ and 353 [Agly+83]^−^ in the MS/MS spectra for peaks 2, 4, 8, 10, 11, and 12, in addition to neutral losses of 60, 90, and/or 120 amu matched the assignment of these peaks as *C*-glycosylated apigenin derivatives [20]. Peak 2 with molecular formula of C_27_H_30_O_15_, deduced from the molecular ions at *m/z* 593.1464 ([M-H]^−^), was fragmented further to give ions at *m/z* 503.1155, for [(M-H)-90]^−^, 473.1051 for [(M-H)-120]^−^, 383.0739 for [(M-H)-210]^−^ or [Agly+113]^−^, and 353.0638 for [(M-H)-240]^−^ or [Agly+83]^−^. Based on these data, peak 2 was identified as apigenin-6,8-di-*C*-hexose.

Peaks 4 and 8 have the same molecular formula of C_26_H_28_O_14_ based on common molecular ions at *m/z* 563.1359 ([M-H]^−^). Further fragmentation resulted in ions at *m*/*z* 473.1053 for [(M-H)-90]^−^, 443.0949 for [(M-H)-120]^−^, 383.0742 for [(M-H)-180]^−^ or [Agly+113]^−^, and 353.0639 for [(M-H)-210]^−^ or [Agly+83]^-^. The [^0,3^_5_X_8_]^−^ ion at *m/z* 503.1168 was formed from a 0,3” breakage of 8-C-pentoyl unit from the aglycon. Both the [^0,3^_6_X_6_]^-^ ions and [^0,2^_5_X_8_]^−^ contributed to the abundance of the ion at *m/z* 473.1053 for [(M-H)-90]^−^, while the ion at *m/z* 443.0949 ([(M-H)-120]^−^) is the [^0,2^_6_X_6_]^−^ ion arising from 6-C-hexosyl fragmentation of the molecule. Based on all this information, peaks 4 and 8 appeared to be isomers of apigenin-C-hexose-C-pentose. The relative abundance of the fragment ions demonstrated the type of sugar at the *C*-6 position [41] and it is known that substituents at the *C*-6 position are more prone to cleavage in flavone [42]. Relative abundance of *m/z* 473.1053, [(M-H)-90]^−^ and 443.0949 [(M-H)-120]^−^ as results of CID fragmentation of *m/z* 563 isomers were examined in order to identify the C-6 substituents of the two isomers. As shown in Table S1, peaks 4 and 8 have higher abundance of the ion at *m/z* 473 fragment ion compared to the *m/z* 443 fragment ion, indicating that the C-6 position of both compounds is most likely attached to a pentose. Therefore, peaks 4 and 8 were assigned as apigenin-6-C-pentose-8-C-hexose isomers.

Peaks 10 and 11 also have the same molecular formula of C_21_H_20_O_10,_ deduced from their molecular ion at *m/z* 431.0947 ([M-H]^−^). Both peaks also showed similar fragmentation pattern, consistently showing the presence of fragment ions at *m/z* 341.0639, 311.0536, and 283.0589, corresponding to [Agly+71]^−^, [Agly+41]^−^, and [Agly+CH_2_]^−^, respectively. Therefore, peaks 10 and 11 were assigned as vitexin (apigenin-8-C-glucoside) and isovitexin (apigenin-6-C-glucoside), respectively, and confirmed by comparing with authentic standards.

Finally, peak 12 showed molecular ion at *m/z* 577.1306 which predicted a molecular formula of C_27_H_30_O_14_. Fragment ions were observed at *m/z* 457.1098 for [(M-H)-120]^−^; 413.0845 for [(M-H)-120-44]^−^, 353.0630 for [(M-H)-120-104]^−^, 341.0640 for [Agly+71]^−^ and 311.0536 for [(M-H)-120-134]^−^. Losses of 44, 74, 104, and 134 amu are characteristic of *C*-deoxyhexose fragmentations [40] and based on the high abundance of the *m/z* 413 fragment ion for loss of one hexose unit, peak 12 being assigned as apigenin-6-C-hexose-8-C-deoxyhexose.

Peaks 1, 3, 5, 6, 7, and 9 were further relatively quantified and expressed as microgram of orientin equivalent per mg dried extract, while peaks 2, 4, 8, 10, 11, and 12 were also relatively quantified and expressed as microgram of vitexin equivalent per mg dried extract (Table 1). Alcohol-based NaDES (BD, GLY, and PD) was able to extract the highest TLC with values ranging from 19.33 to 27.82 µg/mg dried extract, comparable with MeOH (26.16 µg/mg dried extract). The other NaDES (LA, GLU, and MAL) extracted much lower TLC (12.46, 12.02, and 8.73 µg/mg, respectively). In comparison between samples, BD was able to extract and recover the highest amount of luteolin derivatives for each compound except for peak 9 (luteolin-6-C-hexose-8-C-deoxyhexose (Isomer 2) where GLY showed the highest. Furthermore, similar findings were obtained for TAC where alcohol-based NaDES (GLY, BD, and PD) showed superiority in extracting apigenin derivatives with values ranging from 76.06 to 132.79 µg/mg dried extract as compared to LA, GLU, and MAL with TAC values of 43.80, 43.40, and 25.96 µg/mg, respectively. It was discovered that GLY was able to extract the highest amount of apigenin derivatives for compound 8 and 10–12 while BD was better in extracting compound 2 and 4. As a result, GLY showed the highest total apigenin and luteolin content with value of 157.39 µg/mg dried extract followed with BD with value of 141.36 µg/mg. Both NaDES showed higher efficiency than MEOH. This became a good indicator on the high efficiency of tailor-made NaDES to extract flavonoids in particular flavonoid *C*-glycosides. The hydroxyl and carbonyl groups present in the NaDES components may initiate the hydrogen bonding interaction with flavonoid *C*-glycosides in OPL, which have moderate to high polarities. Luteolin and apigenin derivatives shared similar hydroxyl groups (-OH) containing aglycone, which played a significant role in forming an intense hydrogen bond network with NaDES. The NaDES developed at optimized conditions in the present study were suitable for the extraction of flavonoid *C*-glycosides from OPL. The result was consistent with a previous work which also reported alcohol-based NaDES were suitable in extracting apigenin derivatives from *Cajanus cajan* (L.) Millsp [43].

### 3.3. Antioxidant Free Radical Scavenging Activities of NaDES Extracts

The TPC, TFC, DPPH, and NO free radical-scavenging activities of the NaDES extracts are presented in Table 2. The TPCs of alcohol-based NaDES combined with XAD7HP resin was found higher with values ranging from 102.32 to 145.81 mg GAE/g dried extract followed by LA, MAL, and GLU with TPC values of 33.27, 23.96, and 23.30 mg GAE/g, respectively. Meanwhile, the TFC of GLY and BD was slightly lower with values ranging from 26.63 to 28.82 mg QCE/g dried extract as compared to TFC value of MEOH extracts (29.55 mg QCE/g). Moreover, the DPPH free radical scavenging activity of NaDES extracts showed a similar trend to TPC and TFC where alcohol-based NaDES (BD, GLY, and PD) showed the highest DPPH inhibition with values ranging from 93.46% to 94.15% at 100 µg/mL and the result was comparable with MEOH (94.37%). Meanwhile, the NO free radical scavenging activity of BD extract showed the highest NO inhibition with a value of 73.00% while the rest of NaDES extracts showed moderate inhibition with values ranging from 50.62% to 57.34%. This finding indicates the positive relationship of TPC and TFC with the antioxidant activities, especially in DPPH free radical scavenging activity. The combination of alcohol-based NaDES with XAD7HP resin produced extracts that have more potent antioxidant activity and comparable with MEOH extracts. To support this finding, the potential of NaDES in extracting antioxidant compounds has been reported previously from various plant-based materials [11,44,45].

### 3.4. Cell Viablity of OPL–NaDES Extracts

The cytotoxicity effect or cell viability of NaDES extracts was evaluated by MTT assay in 3T3 fibroblast cells. Allantoin was used as positive drug in this study. The cells were exposed to different concentrations of extracts ranging from 1000 to 3.91 µg mL^−1^. As depicted in Figure S3, the extracts showed no toxicity effect on 3T3 cells within tested concentrations indicated by the cell viability percentage reached above 70% except for GLY and BD extracts at 1000 µg mL^−1^ [46]. However, a dose-dependent effect within tested samples whereby the cell viability increased as the extract concentration decreased was observed. At low concentration, the cell viability of PD, GLY, MEOH, and allantoin was found as the highest at a dose of 7.81 µg mL^−1^, BD and LA found at the highest at 3.91 µg mL^−1^, and sugar-based NaDES (GLU and MAL) found the highest at both concentrations 7.81 and 3.91 µg mL^−1^. This result was consistent with previous studies that also found the high cell viability at low concentrations [47,48,49]. It was discussed earlier that the low cell viability at higher concentration is due to the accumulation of compounds onto the cells which could lead to the activation of caspases and apoptosis induction [48,50]. Therefore, 7.81 µg mL^−1^ was selected as a safe dose to be used for scratch assay.

### 3.5. Cell Proliferation and Migration Activity of OPL–NaDES Extracts

A scratch assay was employed to study the cell migration and proliferation activities of NaDES extracts. A linear line was created on the monolayer 3T3 fibroblast cell using a pipette tip (P200) and the progression of wound closure was monitored at 0, 24, and 48 h upon sample treatments. Table 3 shows the visual representation of wound closure in each tested sample prepared at 7.81 µg mL^−1^ including negative and positive controls. There was mild migration and proliferation of cells observed in negative control with 25.46% and 52.30% after 24 and 48 h incubation. Meanwhile, a progressive migration and proliferation of cells was seen in allantoin as positive control with 64.75% and 95.51% after 24 and 48 h incubation, respectively. As indicated in Table 2, the results showed the enhancement of cell proliferation and migration activities of NaDES extracts more than 50% after 24 h of treatment except for GLU extract (37.27%) as compared to negative control. At 48 h, the cells treated with alcohol-based NaDES extracts (GLY, BD, and PD) reached the highest cell migration and proliferation activities with a value higher than 95% as compared to MEOH, LA, MAL, and GLU with values of 93.14%, 91.11%, 89.56%, and 84.72%, respectively. The results are consistent with the significant amount of flavonoid *C*-glycosides found in each extract and their antioxidant activities, suggesting that these antioxidant compounds might play a role in wound repair as was previously described [16,19,21].

### 3.6. Correlation of Flavonoids in OPL–NaDES Extracts with Wound Healing Activities

**Figure 6:**
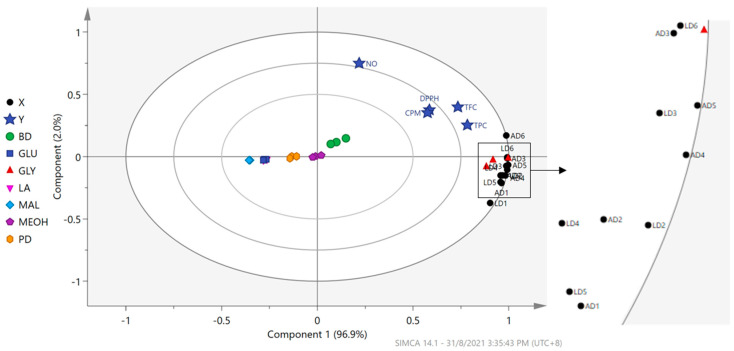
Biplot of the partial least square (PLS) model exhibiting correlation between relatively quantified luteolin and apigenin derivatives and wound healing activity of OPL extracts obtained from various green solvents. Abbreviations: BD, choline chloride–1,4 butanediol; GLU, choline chloride–glucose; GLY, choline chloride–glycerol; LA, choline chloride–lactic acid; MAL, choline chloride–maltose; MEOH, aqueous methanol; PD, choline chloride–1,2 propanediol; TPC, total phenolic content; TFC, total flavonoid content; DPPH, 2,2-diphenyl-1-picrylhydrazyl; NO, nitric oxide; CPM, cell proliferation and migration; LD, luteolin derivatives; AD, apigenin derivatives; LD1, luteolin-6,8-di-C-hexose (Isomer 1); LD2, luteolin-6,8-di-C-hexose (Isomer 2); LD3, isoorientin; LD4, orientin; LD5, luteolin-6-C-hexose-8-C-deoxyhexose (Isomer 1); LD6, luteolin-6-C-hexose-8-C-deoxyhexose (Isomer 2); AD1, apigenin-6,8-di-C-hexose; AD2, apigenin-6-C-pentose-8-C-hexose (Isomer 1); AD3, apigenin-6-C-pentose-8-C-hexose (Isomer 2); AD4, vitexin; AD5, isovitexin; AD6, apigenin-6-C-hexose-8-C-deoxyhexose.

To analyze the correlation between the relative quantities of the flavonoid and antioxidant activity of the NaDES extracts, a PLS model was fitted to a unit variance-scaled dataset (dimension = 21 × 17, where *X* variables = 12 (relative quantity of 12 flavonoids) and *Y* variables = 5 (TPC, TFC, DPPH and NO radical-scavenging and CPM-cell proliferation and migration assays). A two-component PLS model was obtained, with *R^2^X* (cumulative up to component 2), *R^2^Y* (cumulative up to component 2), and *Q^2^* (cumulative up to component 2) of 100%, 90.3%, and 83.8%, respectively. The model was cross-validated following the seven-fold cross-validation procedure. The cross-validation plots of the model with 200 times permutation tests (Figure S4) indicated that the model did not overfit the data. As depicted in Figure 6, the first component (explained variation = 96.9%) of the PLS model showed that the clustering of the NaDES extracts was mainly influenced by the type of HBD composed in the solvents, where samples extracted using GLY were seen farthest at the positive side of the plot whereas BD extracts were also located on the same side but near to the origin of the plot. Meanwhile, samples extracted using MAL, GLU, PD, and LA were positioned at the negative side of the plot. Samples extracted by MEOH were accumulated at the origin of the plot. All X (LD1-6 and AD1-6) and Y (TPC, TFC, DPPH, NO, and CPM) variables were projected at the positive side of the first component, close to the GLY and BD extracts, revealing that these NaDES, particularly GLY, are the best NaDES to extract natural wound healing agents from oil palm leaves.

## 4. Conclusions

The extraction of flavonoids, specifically luteolin and apigenin derivatives from OPL, using natural deep eutectic solvents was investigated. Various choline chloride-based NaDES were formulated with several HBDs (1,2 propanediol, 1,4-butanediol, glycerol, glucose, maltose, and lactic acid), and optimized with respect to method of synthesis, HBA:HBD molar ratio, and water content. Different synthesis methods, molar ratios, and water contents greatly influenced the NaDES capacity to extract optimal TFC. Based on TFC values, the optimal conditions for synthesizing NaDES with high extractability for OPL flavonoids were shown to be PD (SDH, 1:4, 38%), BD (SDH, 1:4, 33%), GLY (SDS, 1:3, 29%), GLU (SWH, 1:4, 50%), MAL (SDS, 1:3, 50%), and LA (SDS, 1:3, 17%). UHPLC-UV/PDA-MS/MS analysis revealed GLY is the most efficient in extracting flavonoid *C*-glycosides, comparable with conventional solvents. These eutectic solvents were successfully employed and integrated with UAE and macroporous resin in a simple and rapid extraction and enrichment process of flavonoids from OPL. The NaDES extracts produced demonstrated a dose-dependent manner on cytotoxicity profile and possessed good free radical scavenging and wound healing activities. In conclusion, the findings of the present study contribute to the possibility of developing an eco-friendly, rapid, and efficient extraction process to produce high quality extracts from biomass using green solvent, which could potentially contribute to the sustainability of the oil palm industry including chemical, nutraceutical, and pharmaceutical industries.

## Figures and Tables

**Table 1:** Putative identification and relative quantification of flavonoids present in oil palm leaf–natural deep eutectic solvent extracts using UHPLC-UV/PDA-MS/MS analysis.

Peak	tR (min)	λmax, (nm)	[M-H]^−^ (*m/z*)	Formula	Key MS/MS Fragments (*m/z*)	Compound	Relative Quantification (µg/mg Dried Extract)						
							PD	BD	GLY	GLU	MAL	LA	MEOH
1	3.17	272, 348	609.1411	C_27_H_30_O_16_	519.1104 489.0998, 429.0786, 399.0696, 369.0585	Luteolin-6,8-di-C-hexose (Isomer 1)	1.55 ± 0.15	1.65 ± 0.06	1.19 ± 0.15	1.21 ± 0.11	1.12 ± 0.07	1.16 ± 0.06	1.88 ± 0.21
2	4.88	272, 336	593.1464	C_27_H_30_O_15_	503.1155, 473.1051, 383.0739, 353.0638	Apigenin-6,8-di-C-hexose	8.36 ± 1.12	10.57 ± 1.26	7.33 ± 0.71	5.68 ± 0.64	4.10 ± 0.42	5.32 ± 0.73	10.90 ± 1.44
3	6.72	272, 346	609.1411	C_27_H_30_O_16_	489.1001, 429.0789, 399.0679, 369.0604	Luteolin-6,8-di-C-hexose (Isomer 2)	1.11 ± 0.04	1.33 ± 0.07	1.21 ± 0.05	0.98 ± 0.04	0.91 ± 0.01	0.97 ± 0.02	1.19 ± 0.05
4	7.10	272, 334	563.1359	C_26_H_28_O_14_	473.1053, 443.0949, 383.0742, 353.0639	Apigenin-6-C-pentose-8-C-hexose (Isomer 1)	2.10 ± 0.11	2.73 ± 0.64	2.36 ± 0.33	1.92 ± 0.02	1.65 ± 0.04	1.82 ± 0.13	2.47 ± 0.16
5	7.86	270, 348	447.0896	C_21_H_20_O_11_	357.0588, 339.0480, 327.0483, 297.0379, 285.0381	Luteolin-6-C-hexose (Isoorientin)	7.18 ± 0.48	11.16 ± 0.83	9.63 ± 0.83	3.87 ± 0.15	2.35 ± 0.06	4.11 ± 0.21	10.32 ± 0.76
6	9.00	270, 350	447.0896	C_21_H_20_O_11_	357.0587, 339.0476, 327.0485, 297.0378, 285.0380	Luteolin-8-C-hexose (Orientin)	4.57 ± 0.73	6.88 ± 1.50	6.09 ± 1.38	2.59 ± 0.49	1.81 ± 0.24	2.84 ± 0.38	6.26 ± 1.18
7	9.87	270, 348	593.1464	C_27_H_30_O_15_	473.1049, 429.0792, 369.0590, 357.0589, 327.0485	Luteolin-6-C-hexose- 8-C-deoxyhexose (Isomer 1)	2.57 ± 0.26	3.42 ± 0.56	2.89 ± 0.38	1.69 ± 0.17	1.32 ± 0.11	1.69 ± 0.15	3.64 ± 0.42
8	11.22	274, 334	563.1359	C_26_H_28_O_14_	503.1168, 473.1056, 443.0950, 383.0743, 353.0639	Apigenin-6-C-pentose-8-C-hexose (Isomer 2)	2.68 ± 0.22	3.57 ± 0.51	3.75 ± 0.61	2.01 ± 0.18	1.66 ± 0.06	2.06 ± 0.17	3.13 ± 0.52
9	11.60	272, 336	593.1464	C_27_H_30_O_15_	473.1067, 413.0846, 369.0590, 357.0589, 293.0434	Luteolin-6-C-hexose- 8-C-deoxyhexose (Isomer 2)	2.36 ± 0.31	3.37 ± 0.29	3.60 ± 0.33	1.68 ± 0.12	1.22 ± 0.07	1.68 ± 0.13	2.87 ± 0.23
10	12.44	270, 338	431.0947	C_21_H_20_O_10_	341.0639, 323.0529, 311.0536, 283.0589	Apigenin-6-C-hexose (Vitexin)	10.23 ± 0.44	14.46 ± 0.66	15.81 ± 0.78	6.24 ± 0.34	4.01 ± 0.17	6.68 ± 0.24	13.89 ± 0.32
11	13.85	270, 338	431.0947	C_21_H_20_O_10_	341.0638, 323.0536, 311.0536, 283.0588	Apigenin-8-C-hexose (Isovitexin)	19.76 ± 0.33	29.43 ± 0.40	32.39 ± 1.97	11.43 ± 0.28	6.58 ± 0.17	11.76 ± 0.70	27.40 ± 0.91
12	17.19	270, 338	577.1306	C_27_H_30_O_14_	457.1098, 413.0845, 353.0630, 341.0640, 311.0536, 293.0432	Apigenin-6-C-hexose-8-C-deoxyhexose	32.93 ± 3.70	52.77 ± 5.94	71.15 ± 8.24	16.12 ± 1.72	7.95 ± 0.77	16.15 ± 1.68	45.08 ± 4.77

Values are presented as mean ± standard deviation of three measurements. Peak assigned to luteolin or apigenin derivatives was quantified relatively in orientin or vitexin equivalents (µg/mg dried extract), respectively. PD, choline chloride-1,2 propanediol; BD, choline chloride-1,4 butanediol; GLY, choline chloride-glycerol; GLU, choline chloride-glucose; MAL, choline chloride-maltose; LA, choline chloride-lactic acid; MEOH, aqueous methanol.

**Table 2:** Polyphenolic contents, antioxidant activities, and wound healing properties of oil palm leaf-natural deep eutectic solvent extracts.

NaDES	Polyphenolic Contents				Antioxidant Activities (100 µg mL^−1^)		Wound Healing Properties at 7.81 µg mL^−1^		
	TPC (mg GAE/g)	TFC (mg QCE/g)	TAC (µg VE/mg)	TLC (µg OE/mg)	DPPH (%)	NO (%)	Cell Viability (%)	Cell Proliferation and Migration (%)	
								24 h	48 h
GLY	145.81 ± 4.11	28.82 ± 0.39	132.79 ± 12.42	24.60 ± 2.33	93.46 ± 3.82	56.16 ± 2.82	231.65 ± 22.55	57.82 ± 2.52	95.52 ± 0.61
BD	105.21 ± 4.99	26.63 ± 2.26	113.54 ± 8.76	27.82 ± 2.59	94.15 ± 4.52	73.00 ± 7.53	225.53 ± 26.09	56.79 ± 1.48	95.02 ± 0.99
PD	102.32 ± 5.52	13.55 ± 2.16	76.06 ± 5.74	19.33 ± 1.24	93.46 ± 2.87	57.34 ± 3.12	278.66 ± 68.08	55.82 ± 5.17	95.43 ± 1.02
LA	33.27 ± 8.14	3.88 ± 0.22	43.8 ± 3.36	12.46 ± 0.67	67.75 ± 2.49	54.15 ± 5.49	247.24 ± 36.86	56.34 ± 5.04	91.11 ± 0.81
MAL	23.96 ± 2.48	2.24 ± 0.62	25.96 ± 1.39	8.73 ± 0.39	72.17 ± 1.78	50.62 ± 5.86	283.82 ± 14.39	51.70 ± 3.83	89.56 ± 2.40
GLU	23.30 ± 3.59	1.29 ± 0.09	43.4 ± 2.69	12.02 ± 0.78	69.26 ± 7.29	51.76 ± 5.99	254.38 ± 47.85	37.27 ± 4.59	84.72 ± 2.04
MEOH	129.64 ± 7.39	29.55 ± 0.31	102.87 ± 7.58	26.16 ± 1.80	94.37 ± 3.10	67.37 ± 1.25	306.72 ± 11.13	63.64 ± 4.58	93.14 ± 0.62

PD, choline chloride–1,2 propanediol; BD, choline chloride–1,4 butanediol; GLY, choline chloride–glycerol; GLU, choline chloride–glucose; MAL, choline chloride–maltose; LA, choline chloride–lactic acid; MEOH, aqueous methanol.

**Table 3:** Representative cell proliferation and migration activities in vitro scratch wound assay at 7.81 µg mL^−1^.

	Control	BD	PD	GLY	GLU	MAL	LA	MEOH	Allantoin
0 h	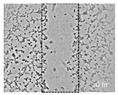	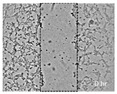	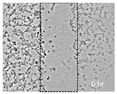	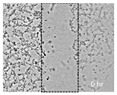	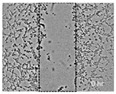	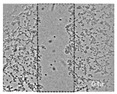	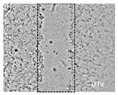	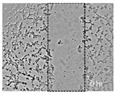	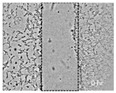
24 h	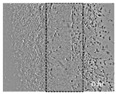	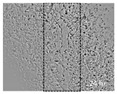	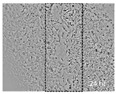	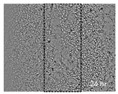	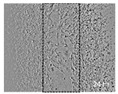	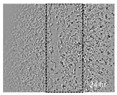	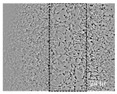	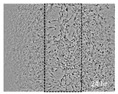	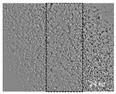
48 h	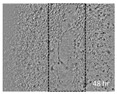	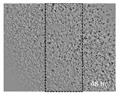	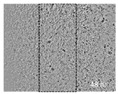	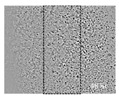	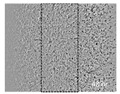	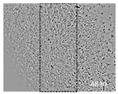	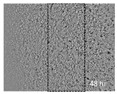	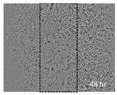	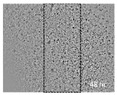

PD, choline chloride–1,2 propanediol; BD, choline chloride–1,4 butanediol; GLY, choline chloride–glycerol; GLU, choline chloride–glucose; MAL, choline chloride–maltose; LA, choline chloride–lactic acid; MEOH, aqueous methanol.

## Data Availability

The data presented in this study are available in this manuscript.

## Supplementary Materials

The following are available online at https://www.mdpi.com/article/10.3390/antiox10111802/s1, Figure S1: Representative of UHPLC-UV/PDA chromatogram of NaDES extracts at 366 nm, Figure S2: Proposed fragmentation pathway of compound 1 and 3, Figure S3: Effect of OPL–NaDES extracts on viability of 3T3 fibroblast cells, Figure S4: Cross-validation plots of PLS model with 200 times permutation tests. Plot for Y-variable TPC (A); TFC (B); DPPH (C); NO (D); and CPM—cell proliferation and migration (E), Table S1: MS/MS Spectra of *m/z* 563 isomers with fragment ion relative abundance information.
