# Supplementary material for: Integration of Choline Chloride-Based Natural Deep Eutectic Solvents and Macroporous Resin for Green Production of Enriched Oil Palm Flavonoids as Natural Wound Healing Agents
Source: Antioxidants (Basel). 2021 Nov 12;10(11):1802. doi: 10.3390/antiox10111802 (PMC8615239; doi:10.3390/antiox10111802)
Supplement: Supplementary file 1 [file antioxidants-10-01802-s001.zip › antioxidants-1424516-supplementary.pdf]

Supplementary materials

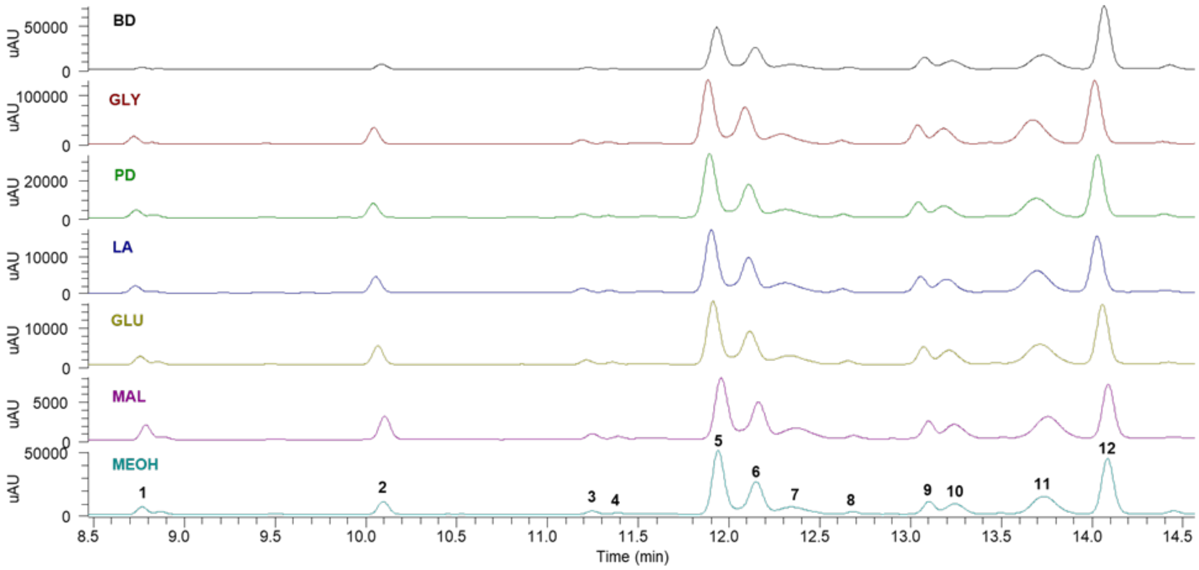

Figure S1 Representative of UHPLC-UV/PDA chromatogram of NaDES extracts at 366 nm

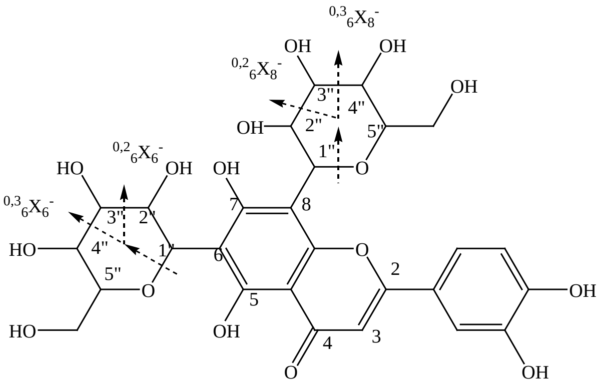

Figure S2 Proposed fragmentation pathway of compound 1 and 3

Table S1 MS/MS Spectra of m/z 563 isomers with fragment ion relative abundance information

Fragment ion	Peak 4	Peak 8
563 $[M-H]^-$	100.0	100.0
473 $[M-90-H]^-$	29.94	44.42
443 $[M-120-H]^-$	25.55	37.03
C-6 Sugar	Pentose	Pentose
Proposed structure	Apigenin-6-*C*-pentose-8-*C*-hexose	Apigenin-6-*C*-pentose-8-*C*-hexose

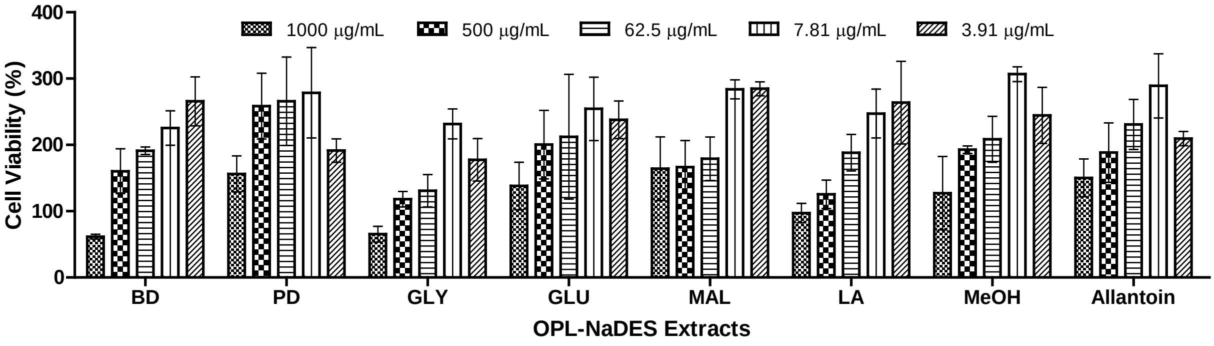

Figure S3 Effect of OPL-NaDES extracts on viability of 3T3 fibroblast cells. Values are presented as mean ± standard deviation of three measurements. PD, choline chloride-1,2 propanediol; BD, choline chloride-1,4 butanediol; GLY, choline chloride-glycerol; GLU, choline chloride-glucose; MAL, choline chloride-maltose; LA, choline chloride-lactic acid; MEOH, aqueous methanol.

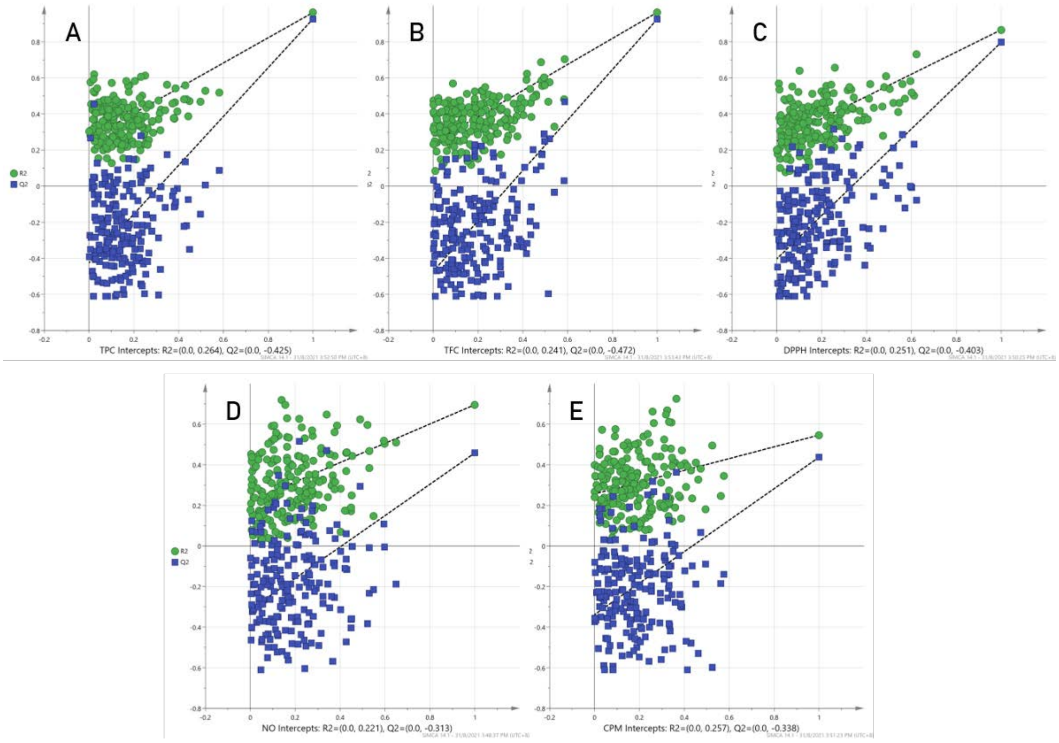

Figure S4 Cross-validation plots of PLS model with 200 times permutation tests. Plot for Y-variable TPC (A); TFC (B); DPPH (C), NO (D) and CPM- Cell proliferation and migration (E)
